# Supplementary material for: Are carbon emissions trading and green financial instruments synergistic? -Comprehensive quantitative research based on content analysis
Source: PLoS One. 2024 Mar 7;19(3):e0298601. doi: 10.1371/journal.pone.0298601 (PMC10919601; doi:10.1371/journal.pone.0298601)
Supplement: S1 Data — (ZIP) [file pone.0298601.s001.zip › Data/地级市Z_F工作报告.zip!/║┌┴·╜¡/║┌║╙╩╨/└┤╫╘╣1⁄2╓┌║┼í╛┬φ┐╦╩2╛▌═°í┐_2006.docx]

各位代表：

现在，我代表市人民政府，向大会报告工作，请予审议，并请各位政协委员和列席会议的同志提出意见。

一、2005年工作回顾和“十一五”时期规划重点

2005年，在省委、省政府和市委的正确领导下，紧紧围绕市三届人大四次会议确定的目标，克服诸多困难和不利因素，经济建设和社会事业较快发展，顺利完成了“十五”计划，为“十一五”规划的制定和实施奠定了坚实基础。全市生产总值实现120.2亿元，同比增长10.8%，与2000年相比，五年增长55.3%，年递增9.2%。城镇固定资产投资26.7亿元，增长13.3%，五年增长65.5%，年递增10.6%。社会消费品零售总额实现33.3亿元，同比增长10.2%，五年增长53.2%，年递增8.9%。财政一般预算收入3.1亿元，同比增长15.1%，五年增长34.5%，年递增6.1%。城镇人均可支配收入5041元，同比增长18.5%，五年增长62.5%，年递增10.2%；农民人均纯收入3324元，同比增长15.5%，五年增长53.9%，年递增9%。

&shy;——农业经济发展态势良好。农业增加值实现29.5亿元，增长18.8%。种植业实现增加值23.5亿元，增长20.8%。粮豆薯总产171万吨，增长16.1%，为历史上最好年份。经济作物播种面积48.56万亩，增长20.4%。绿色食品监测面积达到260万亩，增长30%。大牲畜存栏 24.9万头, 增长15.2%。畜牧业实现增加值3.7亿元，增长15.1%。造林面积17.2万亩。开发境外土地10万亩。输出农村劳动力13.5万人，实现劳务收入5亿元。

——工业经济继续快速增长。工业经济各项指标达到历史最好水平。全市规模以上工业企业增加值实现6.1亿元，增长17.2%，超省下达目标9.2个百分点。地方规模以上工业企业增加值 实现4.4亿元，增长21%，实现利税1.34亿元，增长43.3%，超省核定目标34.8个百分点。效益综合指数为84.5，提高29.7个百分点。国有工业企业改革继续深化，改制面达到96.3%。非公有制经济增加值实现23.6亿元，增长24.2%，占GDP比重达到27.1%。

——对外经贸旅游再创新高。全市对外贸易进出口总值完成5.83亿美元，增长101.7%，实现税收2000万元，增长173%。口岸出入境人数和过货量分别达到92万人次和44.4万吨，增长46.5%和19.3%。接待国内外旅游者94万人次，其中边境旅游30万人次，分别增长17%和54.4%，边境旅游收入1.1亿元，增长13.1%。招商引资实际到位资金12.1亿元，增长74.9%，引进超千万元以上项目36项，到位资金8.1亿元，增长69.3%。实际利用外资1182万美元，增长80.7%。

——城乡建设步伐明显加快。完成了各县（市）区重点区域、城区供热系统详控规划编制和黑河市区地形图测绘工作。全市投入1.8亿元进行城市基础设施建设，同比增长36%，其中市区投入1.04亿元，是历年来投入最多的一年，重点推进了城市道路改造、公园建设、王肃街建筑立面改造等9项工程。全市新建住宅51万平方米，增长8.6%，人均住房建筑面积20.2平方米，增长6%。重点城镇的道路、给排水等基础设施进一步完善，新修通县乡和通村公路里程分别达到192公里和784公里。

——重点项目建设成效显著。新兴基础原材料加工区一期、恒基水泥厂、北安电厂三号炉复建项目建成投产。吉斯达商务港展馆、金湾水电站、垃圾处理厂、黑河热电厂三期和嫩江热电厂开工建设。多宝山铜矿开发项目顺利推进。黑龙江公路大桥完成了国内报批手续，引道工程开工建设。对俄购电线路、铁路间油气换装管道等项目取得进展。黑呼、嫩多公路升级改造和黑河港改扩建工程前期工作扎实推进。21个财源项目被省财政厅确定为重点扶持项目。

——财政保障能力得到加强。全口径财政收入5.8亿元，增长25.2%。地方一般性预算支出22. 5亿元，增长29.9%。按现行财政体制计算，全市一般预算收支净结余170万元。重点支出得到保障，农业、社会保障、科教等方面支出大幅增加。多年来困扰我市的工资发放和运转问题基本得到解决，财政运行质量有所提高，财政工作重点逐步转移到支持经济和社会发展上来。

——社会事业全面发展。实施市级以上科技发展项目55项，其中省级以上立项20项，高新技术产业产值和民营科技企业产值均实现两位数增长。小学适龄儿童、初中适龄少年入学率分别达到100%和99%，小学和初中辍学率分别控制在0.11%和0.85%，分别低于省控制指标0.89和2.15个百分点。全市6个传染病区项目投入使用，社区卫生服务建设和爱国卫生创建达标率均为100%。北安市农村合作医疗试点工作稳步推进。文化体育事业日趋繁荣，群众文体活动蓬勃开展。人口自然增长率控制在4.65‰以下。“四五”普法通过省验收，“平安黑河”建设取得明显成效。其他社会事业也取得新进展。

——人民生活水平逐步提高。城镇新就业22422人，登记失业率4.04%，比省控制指标低1.46个百分点。职工工资和三条保障线资金发放率均为100%。完成了4.7万名国企下岗职工向失业保险并轨工作，失业保险参保人数已达6万余人，医疗保险参保人数11.3万人。城乡居民储蓄存款余额130亿元，比年初增长10.9%。市区和农村人均住宅面积分别达到25.4、18.5平方米，农村住宅砖瓦化率达到62.2%。

——政府自身建设进一步加强。深入贯彻《行政许可法》，行政许可部门由63个减少到55个，行政许可项目由463项减少到257项。全面推行政务公开和“六项制度”，加强行政效能监察，行政审批事项由147项减少到129项。坚持开展先进性教育、公务员培训和党风廉政建设，政府行政能力不断提高。

一年来，我们坚持牢牢把握第一要务，聚精会神搞建设，一心一意谋发展，积极推进结构调整，实施项目牵动战略，加大招商引资和向上争取力度，着力解决好关系全局和长远发展的重大问题，努力保持国民经济快速、健康发展的良好势头；坚持深化改革开放，注重用改革的举措破解难题，大力发展非公有制经济，不断提高黑河对外开放水平；坚持以人为本，着力解决事关人民群众切身利益的就业、社会保障、衣食住行等方面问题，为人民群众办好事、办实事，化解各种矛盾，维护社会稳定，努力构建和谐社会；坚持科学发展观，切实转变政府职能，深入调查研究，科学施政和依法行政，规范行政行为，不断提高施政能力；坚持依靠市委领导，认真执行市委决策，主动接受人大、政协监督，定期报告工作和通报情况，共办结人大代表建议25件，政协委员提案131件，加强了与工会、共青团和妇联等群众团体的联系。

各位代表，2005年的工作成绩来之不易，与省委、省政府和市委的正确领导，与市人大、政协及各民主党派的监督和支持，与中省直部门的大力配合，与驻黑河解放军和武警部队的无私奉献，与全市各族人民的共同努力密不可分。在此，我代表市政府表示崇高的敬意和衷心的感谢！

在总结成绩的同时，我们也清醒地看到，发展中还存在诸多困难和问题。主要是：经济总量小，发展压力大，结构性矛盾突出，经济持续快速增长的基础尚不稳固；农业产业化水平低，农民增收的渠道还不宽；工业化程度不高，结构调整任务艰巨；第三产业发展规模小，旅游业受国家政策影响，出境人数大幅下降；重要产业、重大项目的支撑带动作用不够明显，资源和区位优势尚未充分发挥；各类人才匮乏，科技创新能力弱；一些与群众利益密切相关的热点、难点问题解决的还不够好。政府部门的一些工作人员思想不够解放、服务意识不强、办事效率不高，发展环境还不宽松，等等。对此，我们将高度重视，采取有效措施，认真加以解决。

各位代表，“十一五”时期是全面建设小康社会的关键时期。按照市委三届三次全会部署，“十一五”时期工作指导思想是，以科学发展观和构建和谐社会为统领，以产业发育为主线，以项目建设为支撑，以改革开放和科技进步为动力，以提高人民生活水平为根本出发点，推进对俄经贸科技合作战略升级，夯实农业发展基础，壮大地方工业实力，建设一个开放地位突出、产业经济发达、发展环境优良、人民生活富裕、社会文明和谐的新黑河。预期目标是，地区生产总值年递增10%以上，城镇固定资产投资年递增20%以上，全口径财政收入年递增9%以上，城镇居民人均可支配收入和农民人均纯收入年分别递增10%和5%，单位生产总值能耗比“十五”期末降低20%左右。主要任务是，全方位推进对俄经贸科技合作战略升级；以资源开发和产品加工为重点，大力发展原材料、能源、绿色食品、林木、医药、化工六大产业，推进新型工业化进程；以工补农，以城带乡，发展现代农业，加快建设社会主义新农村；以旅游业为重点，提升传统服务业档次和服务水平，大力发展现代服务业；加强交通和城市基础设施建设，增强对经济社会发展的支撑和保障能力；推进生态市建设，发展循环经济，建设资源节约型和环境友好型社会；进一步深化体制改革，形成有利于转变经济增长方式、促进全面协调可持续发展的体制和机制；按照建设创新型社会要求，培育壮大创新型人才队伍，促进科教兴市和人才强市战略深入实施；以富裕人民为根本，统筹经济社会发展，不断提高城乡群众物质文化生活水平和质量，加快建设文明和谐新黑河。建设重点是，建设黑龙江大桥，完善港口、铁路等基础设施，构建对俄经贸大通道。依托优势资源和现实基础，优化产业布局，建设“三带十区”，即：北安—五大连池—嫩江现代农业产业带、嫩江—爱辉—逊克矿产开发产业带、五大连池风景区—孙吴二战遗迹—黑龙江（跨国）旅游带；黑河新兴基础原材料加工区、黑河对俄进出口加工区、黑河吉斯达国际物流区、黑龙江大桥桥头区、黑河三吉屯煤电化循环经济区、多宝山矿业开发区、五大连池风景区综合开发区、逊克中俄合作加工区、北安工业区、嫩江九三工业园区，强力推进产业扩张，培育新的经济增长极，实现黑河快发展、大发展。

二、2006年工作安排

2006年是“十一五”规划的起步年，我们要坚持以邓小平理论和“三个代表”重要思想为指导，认真贯彻党的十六届五中全会和省委九届七次全会精神，按照市委三届三次全会部署，坚持科学发展观，把握发展主题，依靠改革开放和科技进步，加快经济结构调整步伐，启动新农村建设，壮大工业实力，推进对俄经贸科技合作战略升级，提升第三产业，协调发展社会事业，构建和谐社会，不断提高人民生活水平，努力实现“十一五”规划良好开局。预期目标是，地区生产总值增长8.5%，城镇固定资产投资总额增长13%，社会消费品零售总额增长10%，全口径财政收入增长7%，外贸进出口总额增长20%，城镇居民人均可支配收入和农民人均纯收入分别增长8%和5%。实现上述目标，必须集中精力抓好以下九个方面工作。

（一）促进农民增收，努力实现新农村建设良好开端。围绕社会主义新农村建设，着力发展优势农业、市场农业、生态农业和项目农业，促进农业增效和农民增收，力争农业增加值增长4%。发展壮大优势产业。种植业坚持“打绿色牌、走特色路”，以打造北安—五大连池—嫩江现代农业产业带为核心，积极推进国家大型商品粮基地建设，“双高”大豆种植面积达到520万亩，增长15%。绿色食品监测面积力争达到300万亩，增长15%。畜牧业坚持“主辅换位”不动摇，突出发展“两牛一羊”和特色养殖，提高规模和水平，畜牧业产值占农业总产值的比重达到25%。加强技术服务，抓好禽流感等畜禽疫病防治工作。林业从搞活经营体制入手，抓好嫩江、爱辉两个县（区）林权改革试点，推进林权流转，扩大大果沙棘、柞桑蚕、北药等基地规模，发展林产经济，提高生态效益和经济效益。外向型农业突出抓好大黑河岛中俄农业科技示范园区建设，促进对俄农业科技交流与合作。搞好爱辉、逊克、北安对俄果蔬出口基地建设，提高黑河果蔬批发市场辐射能力，扩大出口规模。推进境外农业开发，力争达到50万亩。转变农业生产方式。推进场县共建，促进农机农技相结合，提高标准化作业和生产管理水平。培育壮大龙头企业，推进20万吨大豆深加工项目，发展粮食、绿色食品、畜产品、山珍产品等加工业，引导企业与农户间建立产销对接和利益联结机制，提高产业化水平。鼓励和扶持农民创办各类合作经济组织，力争达到500个，提高农民组织化和农业市场化程度。引导农民依法、自愿、有偿流转土地，力争流转108万亩，增长10%。抓好劳务输出，转移劳动力14万人。强化基础设施配套。认真落实50个农机合作社组建计划，提高农机综合作业水平。搞好农业综合开发、农田水利工程、国家级节水灌溉等项目建设，抓好水土流失治理和生态环境保护，改善农业生产条件。加快推进象山水库消险加固、城区堤防配套和防洪排涝工程建设，增强抗灾避险能力。加快实施农村人畜饮水安全工程，力争解决80个村屯饮水难问题。推进服务体系建设。搞好农业综合服务体系和农业远程培训工程建设，鼓励科研单位、农业院校参与农业技术服务，增强为“三农”服务的整体功能。深入实施良种工程，加快良种引进、培育和推广，促进良种更新。推广使用先进技术，完善生产技术规程，健全质量检验监测体系。加快推进龙镇粮食销售、爱辉果蔬批发、北安畜牧交易等专业市场体系建设，促进农副产品流通销售。加强农用生产物资市场监控，维护农民利益。抓好新农村建设试点。注重试点带动，实行分批推进，着力抓好6个乡（镇）、64个村试点启动工作。认真做好规划编制工作，做到规划到乡、到村。立足实际谋划发展项目，并与国家和省有关政策对接。抓好培训工作，培养有文化、懂技术、会经营的新型农民。加强农村路、水、电、讯等基础设施建设，大力发展农村各项社会事业，改善生产生活条件。充分调动农民参与新农村建设积极性，扩大公共财政覆盖农村的范围，积极争取上级扶持，形成多元化投入长效机制。三年完成农业普查工作。

（二）壮大园区经济和重点产业，推进新型工业化进程。以工业园区为重要载体，加快新型工业化步伐，不断提高工业经济总量和运行质量。做大做强园区经济。按照规划先行、全面启动、重点突破的要求，以新兴基础原材料加工区、黑河对俄进出口加工区、三吉屯煤电化循环经济区和多宝山矿业开发区为重点，做好总体规划和产业规划，超前完善基础设施，建立健全服务体系，吸引各类投资主体入区兴业，带动全市工业经济向规模化、集群化发展。推进矿产资源开发。广泛吸引社会资金，加快资源勘查进度，进一步做好多宝山、五大连池、三吉屯和东安等区域的地质勘探工作，解决长期以来制约资源开发的瓶颈问题。引入市场竞争机制，严格按照法定程序招投标和拍卖矿业权，保证矿产资源开发健康有序推进。加大多宝山铜矿、翠宏山铁多金属矿、东安和三道湾子岩金矿等项目的开发力度，筹建关鸟河水泥厂。黑宝山、宋集屯煤矿提高回采率，延长生产年限。151煤矿进一步挖潜提效，富宏、同工煤矿达产，兴边、东兴煤矿年内投产。抓紧研究三吉屯煤田综合开发，实行煤电、煤化一体联动，发展煤化工产业，筹建10万千瓦劣质褐煤综合利用发电厂。继续推进40万吨矿泉水开发项目，启动“矿泉城”建设。发展循环经济。以提质降耗为目标，重点推进黑河热电厂三期、嫩江热电厂、嫩江啤酒厂、北安进发塑料制品有限公司、北安动物制药厂技术改造项目，争取年内投产或达产。争取新兴基础原材料加工区、多宝山矿业开发区、黑河热电厂粉煤灰综合利用项目列入省循环经济试点园区或试点项目。深化国企改革。推进西岗子煤矿重组、西沟水电有限公司政府股份退出、逊克库尔滨水电公司改制、黑河热电厂及五大连池山口水电公司经营方式改革。加强运行监控。抓好重点行业、重点企业、重点产品监测分析和预测预警，有效指导产销衔接。与银行、铁路等部门搞好协调对接，做好资金、运输等生产要素的保障。加大扭亏增盈工作力度，降低企业亏损面和亏损额。

（三）发挥比较优势，不断提高对俄经贸科技合作水平。以中国“俄罗斯年”活动为契机，打造“两国一城”，充分发挥平台作用，推进对俄经贸科技合作战略升级。强化通道设施建设。加强对俄协调，力争黑龙江大桥主体工程年内开工建设。改造黑河口岸货检码头，年内启动滚装码头及铁路专用线建设，提高港口吞吐和铁路运载能力。加快推进逊克口岸基础设施建设，增强服务功能。引进扶强经贸主体。强化内引外联，创造条件吸引过货额度大、带动能力强的经营主体从事对俄经贸合作。同时，充分利用国家和省出台的优惠政策，鼓励和支持现有经贸企业发展壮大，力争全市有业绩的经贸企业达到160家，贸易额超亿美元企业达到3—5家，超千万美元企业达到8—10家。拓展合作领域。实施“走出去”战略，进一步加强与俄罗斯在能源、矿产、林业等方面合作。建设黑河—布市铁路间油（气）换装管道项目。扩大对俄购电规模，力争达到10亿度。扩大在俄木材采伐和建筑工程承包规模，争取分别达到50万立方米和20万平方米。特别是在对俄矿产资源开发方面争取有突破性进展。完善政府间协商联动机制，推动两地合作由经贸向科技、教育、卫生、文化等领域拓展，办好旅游文化交流活动。加强与俄阿州、布市之外的区域合作，推动贸易领域向俄罗斯腹地延伸。调整优化贸易结构。在巩固互市贸易、稳定发展边境贸易的基础上，突出发展一般贸易，扩大自营产品出口规模和代理范围。提高进出口商品档次，稳定已有骨干商品进出口规模，扩大俄化工原料、基础原材料进口和机电、家电等高附加值商品出口。推进加工基地建设。瞄准俄罗斯及国内市场和资源，结合黑河对俄进出口加工区和北安工业区建设，建立生产加工基地，尽快形成规模，拉动就业和税收。鼓励有条件的企业，利用整机与部件的出口税差，发展跨境出口加工业。

（四）以发展旅游业为重点，提高第三产业整体水平。完善服务设施，提高服务功能，壮大产业规模。突出发展旅游业。进一步完善《黑河市旅游业十一五发展规划》，完成重点旅游项目详控规划。实施精品战略，精心设计和培育五大连池—孙吴二战遗迹—黑龙江界江（跨国）旅游带，推出火山游、风情游、知青返乡游等特色旅游产品。积极推进五大连池风景区综合开发，做好国家自然遗产和火山之乡申报及自然保护区立法工作，办好五大连池火山旅游节。加快推进黑河中俄民族风情园等景点建设，做好沾河、库尔滨河、法别拉河等漂流项目提档升级和五大连池风景区4A级景区申评、瑷珲历史陈列馆3A级景区争取工作。引资建设星级宾馆，加快推进五大连池旅游接待中心建设。抓好首次出境游优惠政策落实，强化宣传推介和外联促销，激活旅游市场，力争接待国内外游客百万人次。加快发展现代服务业。培育骨干物流企业，积极发展第三方物流，构筑连接内外、辐射俄罗斯的物流网络。加快推进吉斯达国际商务港建设，增强商务会展、信息咨询、商务洽谈、货运物流等服务功能。推进中心市场和专业批发市场建设，提高物流效能。多方投入，积极发展连锁经营、物流配送、电子商务、特许经营等现代营销方式和新型流通业态。加快发展金融保险、信息咨询等现代服务业。繁荣传统服务业。以市场为导向，以企业为主体，运用现代经营方式和服务技术，改造提升商贸流通、交通运输、餐饮服务等传统行业。抓紧制定商贸业布局规划，针对俄罗斯人消费特点谋划建设专业服务区。培育试车经济，带动相关服务业。积极发展社区服务业。

（五）围绕大项目牵动战略，强力推进招商引资工作。依靠优势项目扩大招商引资，通过大上项目、上大项目，推动黑河经济快发展、大发展。加强项目库建设。根据市场需求、国家产业政策和省确定的投资方向，做好项目谋划工作，重点抓好矿产、能源、旅游、农业产业化、基础设施以及重大社会事业项目的储备，做深做细前期要件，达到招商引资和争取贷款的标准和条件。加快重点项目推进步伐。除抓好前面提到的重点项目外，积极推进布星线和扎逊线220千伏输变电工程、金湾水电站、北安热电有限公司新增5万千瓦发电机组、二门山水电站建设，加快孙吴热电厂、乌二水电站、新鄂水电站前期工作。完成爱辉城区至新生乡、五大连池至讷河、孙吴县城至卧牛河乡公路建设，启动111国道嫩江至讷河段、黑洛公路黑河至呼玛段、209省道嫩呼公路嫩江至多宝山段建设，实施农村公路通达工程和通畅工程，加快道路运输基础设施项目建设。大力开展招商引资。发挥园区设施完善、产业集聚的优势，吸引投资大、牵动力强的项目落户。积极探索有效方式，坚持职能部门招商与全员招商相结合、政府招商与企业招商相结合、重大活动招商与日常招商相结合，提高成功率。以浙江、江苏和香港等地为重点，开展招商活动，组织参加哈洽会以及全省组织的其他招商活动，办好大黑河岛交易会。完善各项制度和措施，强化经费保障。对已签约的重大项目，成立有领导负责的推进组和服务组，提高履约率。力争引进到位资金15.6亿元，增长30%，实际利用外资1400万美元，增长18%。努力壮大非公有制经济。按照“非禁即入、平等对待”的原则，鼓励个人及非公有制企业投资经济建设、社会事业和基础设施建设等各个领域，形成一批新型非公有制骨干企业。扶持和鼓励经营规模较大、带动能力较强的非公有制企业组建企业集团，培育大型龙头企业。认真兑现扶持政策，扩大规模，提高质量，促进非公有制经济快速发展。进一步优化发展环境。在改善服务环境、信用环境和法制环境上下功夫，建设“平安黑河”、“诚信黑河”。及时了解企业的意见和建议，帮助企业解决实际困难和问题，保护客商合法权益，让投资者感到放心、安心、顺心。

（六）遵循发展定位，进一步提升城市整体形象。按照城市规划定位，努力推动城市建设和管理上层次、上水平，不断完善城市功能，提高文化品位，提升国际形象。牢固树立规划第一的观念。始终把规划作为城市建设、管理和发展的第一位工作，建立完善科学民主的规划决策机制。加大规划行政监管力度，杜绝未批先建、违反规划用途等行为。按照适度超前、突出特色的原则，高起点、高标准编制总体规划、详控规划和修建性详细规划，新编详控规划面积90公顷，完成省政府下达的编制任务。坚持以城建城、以城养城的工作思路。抓住国家支持城市基础设施建设的政策机遇，争取建设改造资金。把政府财政信用同市场要素有机结合起来，搭建经营平台，实现多元投入。全面推行经营性土地“招拍挂”工作，增加土地收益。制定相应办法，做到房地产开发与基础设施的建设和完善紧密结合。各县（市）区也要在经营城市方面取得实质进展。加快推进城市基础设施和房屋建设。城市基础设施建设通过多元投入，补还历史欠帐。市区投入1.5亿元推进绿化、道路、公园、中央商业步行街、垃圾处理厂等8项重点工程，年内完工。科学制定市区西部平房区改造规划，结合港口、铁路扩能，加快启动改造项目，争取3年内完成基础设施建设，治理城市污染源，改善人居环境。把住新建工程质量关，加强监理，确保质量。努力提高城市管理水平。按照依法管理城市的要求，理顺城管体制机制，全面推行目标管理责任制，实行精细管理，提高城市管理效能。深入开展“无烟城市”创建和以治理“脏、乱、荒”为重点的环境整治工程，为群众创造一个安全、卫生、便利、舒适的环境。抓好市政公用行业的各项改革。推进物业管理产业化步伐，逐步扩大物业管理的覆盖面。

（七）做好财税金融工作，为经济社会发展提供财力保障。广辟渠道，开源节流，努力增加收入，保证重点支出，确保收支平衡。强化税收征管。建立经营业户数据管理、税源登记、税费征缴网络管理中心，在突出抓好骨干税源征管的同时，切实加强零散税种征收。继续开展定额复查和漏征漏管户清理，严格依法治税，做到应收尽收。认真执行收支脱钩和收缴分离制度，强化监管稽查，确保非税收入增长10%。优化支出结构。按照公共财政要求，严格控制一般性支出，集中财力确保职工工资、离退休人员基本养老金和医疗费、下岗职工基本生活费、城市居民最低生活保障金及时足额发放。 提高市直单位车辆维护费用和办公经费标准，保障党政机关正常运转。保证重点项目、招商引资等前期经费，支持农村基础设施建设和社会事业发展。加快财源建设。下大力气抓好财源项目，对已纳入省重点财源扶持范围的项目全力推进，如期实现达产达效。抓紧谋划论证一批新财源项目，争取更多项目得到上级支持。深化财政体制改革。全面推行国库集中支付制度，不断扩大政府采购范围，加快推进部门预算改革，深化“收支两条线”管理改革，强化预算约束。逐步深化国有资产管理体制改革，搞好公共资产、资源的整合与经营，优化公共资源配置，提高使用效益。加大对财政重点支出项目和专项资金使用的监督检查和审计。加大向上争取力度。围绕资源开发、对俄合作、产业培育和项目建设，研究和把握国家、省产业政策和投资方向，抓住各种有利机遇，千方百计向上争取资金和政策，弥补财力和事业发展资金缺口。做好金融工作。加大金融对经济发展支持力度，创新信贷服务品种，引导和刺激新兴消费。积极做好核呆工作，降低不良资产比例。支持金融业发展，打击逃废债行为，维护金融安全。探索组建地方城市商业银行，改革农村信用社，支持地方经济发展。完善信用担保体系，抓住开发银行支持担保业的契机，使市担保公司资本金达到1亿元以上，为中小企业贷款提供信用服务。

（八）全面发展各项社会事业，推动经济社会协调发展。坚持经济社会统筹发展，在抓经济的同时推动社会全面进步。加强科技和人才工作。积极引进人才、技术和项目，促进科研成果转化。加强对俄科技交流合作，促进资源共享。引人与引智并重，外聘与内培相结合，围绕重点产业和大项目招聘高层次人才，全市行政事业单位3年内招聘储备300名重点本科院校毕业生，为经济社会发展提供人才和智力支撑。以激发人才活力、促进事业发展为目标，积极推进事业单位综合配套改革。优先发展教育事业。巩固“普九”成果，加大基础教育课改力度，提高素质教育水平。大力发展职业教育和成人教育。积极争取黑河学院确立“省市共建、以省为主”的新体制。整合市直属中学教育资源，加快推进黑河中学易地新建，确保如期完工，解决市区初中班额大和第三中学学生上学路途远、条件差、不安全的问题。抓好中小学危房改造，改善教育条件。深化教师聘任制改革，设立财政专项奖励资金，建立教学奖励和激励机制，建设园丁小区，调动广大教师工作积极性。进一步理顺教研管理体制，完善教师培训机制，提高教学质量。大力发展卫生事业。加强公共卫生体系建设，不断提高应对突发公共卫生事件的能力。加强区域中心乡镇卫生院建设，力争完成17个乡镇卫生院改扩建。做好新型农村合作医疗试点和调研工作，扩大社区卫生服务覆盖面，逐步解决群众看病难、看病贵问题。抓好人口与计划生育工作，确保人口出生率控制在9.5‰以内，生育符合政策率达到90%。积极发展文体事业。大力发展文化事业和文化产业，创造更多的优秀文化产品，丰富群众文化生活，特别是活跃农村群众文化生活，推进“边疆文化名城”建设。启动建设市区青少年活动中心和群众艺术馆。规划广播电视中心，加快“村村通”工程建设步伐，提高广播电视人口有效覆盖率。推进黑河有线电视数字化。发展体育事业，开展全民健身活动，提高人民健康水平。加强生态市建设和环境保护工作。坚持在发展中实施保护、在保护中促进发展的原则，大力发展循环经济，推行清洁生产。抓紧制定和实施本地植被恢复、退耕还林还草、防沙治沙、水土保持和自然保护区建设年度计划。认真执行国家有关法律法规，抓好全民环境教育，普及环保知识，开展“绿色单位、绿色学校、绿色社区”创建活动，为建设资源节约型和环境友好型社会奠定坚实基础。同时，进一步做好民族、宗教、外事、侨务、老龄、残疾、审计、统计、档案、气象、文博图、地方史志等项工作。加强国防动员和民兵预备役工作，争创全国“双拥模范城”。

（九）切实关注民生，全心全意为群众办实事。坚持以人为本，从人民群众最关心、最直接、最现实的利益问题抓起，全心全意为群众办实事、解难事、做好事。突出抓好就业和社会保障。认真落实政策，抓好技能培训，努力创造更多就业岗位，城镇登记失业率控制在4.5%以内。继续推进养老保险制度改革，扩大社会保险覆盖面，强化基金征缴。进一步完善失业、医疗、工伤等社会保险制度，增强保障能力。城市居民最低生活保障金月人均补差额由65元提高到70元。完善城乡社会救助体系，帮助困难群众解决实际问题。年内实现每个县（市）区拥有一所服务功能比较完善的农村中心敬老院，提高农村“五保户”集中供养比例。多渠道增加群众收入。巩固农村税费改革成果，落实惠农政策，坚决防止农民负担反弹，增加农民现金收入。全面落实最低工资制度，维护企业职工合法权益。全额发放机关事业单位人员工资，落实公务员各项补贴，适当提高住房公积金、医疗保险金财政匹配比例和供热费补贴标准。加强安全生产管理。认真落实责任制，抓好煤矿生产等行业的安全管理，强化对交通运输、食品安全、易燃易爆品、公共场所的监督检查，完善应急救援预案，消除安全隐患，坚决防止重特大安全事故发生。做好森林防火工作，确保不发生重特大火灾，保证林区居民安全。维护社会稳定。高度重视信访工作，坚持依法治访，妥善处理群众上访问题。引导群众以理性合法的方式表达利益要求，促进社会人际关系的和谐。抓好“五五”普法开局，切实提高公民法律素质。完善防控体系，加强社会治安综合治理，深入开展专项整治行动。加强边境管理，维护边境安定。

三、加强和改进政府工作，不断提高行政能力

树立科学的发展观和正确的政绩观，进一步提高政府行政效能，建设群众满意政府。强化科学发展理念。将科学发展放在首要位置，坚持速度与效益相一致，既要加快发展壮大总量，更要集约发展提高效益；坚持经济发展与环境保护相协调，在加快资源开发的同时，注重发展循环经济，实现可持续发展；坚持城市繁荣与农村发展相促进，统筹城乡发展，加快城镇化进程；坚持经济发展与社会进步相统一，在发展经济的同时，努力建设和谐黑河。进一步解放思想。强化与时俱进、开拓创新、发展是硬道理的思想意识，努力破除影响改革发展的思想障碍和体制障碍，在管理体制、经营体制、经济运行方式等各个方面实现创新。以“三个有利于”为标准，把上级方针政策与黑河市情创造性地结合起来，努力体现黑河工作特色。转变政府职能。加快服务型政府建设，深化行政审批制度改革。贯彻落实《中华人民共和国公务员法》，严格执行《黑河市国家公务员公共服务行为问责暂行规定》，规范公务员行为。加强行政审批服务中心建设，实行“一站式”、“一条龙”综合服务。推进政务信息化建设，优化行政流程，开展政府网上公共服务，提高工作效能。坚持依法行政。认真贯彻落实《全面推进依法行政实施纲要》和《行政许可法》，实施《黑河市依法行政五年规划》，严格实行执法责任制和执法过错追究制，做到有权必有责、用权受监督、侵权须赔偿、违法要追究。规范行政执法行为，切实解决多头执法、执法不公的问题，认真做好行政复议工作。在市委的领导下，认真执行人大及其常委会的决议和决定。自觉接受政协的民主监督，虚心听取民主党派、工商联、无党派人士和各人民团体的意见，确保人民赋予的权力真正服务于人民。推进科学决策。重视民主决策和科学决策，使各项决策更加切合市情、顺应民意。拓宽新闻舆论、社会公众监督等渠道，加快建立和完善重大问题集体决策、专家咨询、社会公示和社会听证制度。今后凡涉及经济社会发展全局和群众切身利益的重大事项，都要进行充分论证和社会听证，让人民群众更广泛地参与公共事务管理。加强廉政建设。坚决贯彻执行反腐倡廉各项规定，全面落实党风廉政建设责任制。充分发挥监察、审计等监管职能，对政府投资项目实行预算审查、公开招投标、过程监管和竣工审计。认真办好“行风视点”节目，继续开展“端正政风行风、优化经济发展环境”最佳最差单位评议活动，坚决纠正部门和行业不正之风，严肃查处违法违纪案件。始终坚持警钟长鸣，廉洁从政，以廉政建设的新成果，进一步树立政府的良好形象。狠抓工作落实。今年是新农村建设的起步之年，也是实施“十一五”规划、促进黑河经济社会全面发展的开局之年，各项工作十分繁重。各级政府、各部门特别是领导干部，要以求真务实的作风，深入基层，调查研究，大力推进，加强督办，把各项任务真正落到实处。

各位代表，黑河新一轮发展的宏伟蓝图鼓舞人心，催人奋进。让我们在市委的正确领导下，团结全市各族人民，进一步抢抓机遇，锐意进取，勇于创新，扎实工作，为实现“十一五”规划良好开局、推进黑河经济健康发展和社会事业全面进步而努力奋斗！
